# Environmental Determinants of Bicycling Injuries in Alberta, Canada

**DOI:** 10.1155/2012/487681

**Published:** 2012-11-28

**Authors:** Nicole T. R. Romanow, Amy B. Couperthwaite, Gavin R. McCormack, Alberto Nettel-Aguirre, Brian H. Rowe, Brent E. Hagel

**Affiliations:** ^1^Departments of Paediatrics and Community Health Sciences, Alberta Children's Hospital, University of Calgary, 2888 Shaganappi Trail NW, Calgary, AB, Canada T3B 6A8; ^2^Department of Emergency Medicine and School of Public Health, University of Alberta and 1G1.50 Walter Mackenzie Centre, 8440–112 Street, Edmonton, AB, Canada T6G 2B7; ^3^Department of Community Health Sciences, University of Calgary, 3rd Floor TRW Building, 3280 Hospital Drive NW, Calgary, AB, Canada T2N 4Z6

## Abstract

This study examined environmental risk factors for bicycling injuries, by combining data on bicyclist injuries collected by interviews in the emergency department (ED) with street-level environmental audits of injury locations, capturing path, roadway, safety, land use, and aesthetic characteristics. Cases were bicyclists struck by a motor vehicle (MV) or with severe injuries (hospitalized). Controls were bicyclists who were not hit by a car or those seen and discharged from the ED, matched on time and day of injury. Logistic regression odds ratios (ORs) adjusted for age, sex, peak time, and bicyclist speed with 95% confidence intervals (CIs) were estimated to relate injury risk to environmental characteristics. Factors contributing to MV events included greater traffic volume (OR 5.13; 95% CI [1.44, 18.27]), intersections (OR 6.89; 95% CI [1.48, 32.14]), retail establishments (OR 5.56; 95% CI [1.72, 17.98]), and path obstructions (OR 3.83; 95% CI [1.03, 14.25]). Locations where the road was in good condition (OR 0.25; 95% CI [0.07, 0.96]) and where there was high surveillance from surrounding buildings (OR 0.32; 95% CI [0.13, 0.82]) were associated with less severe injuries. These findings could be used by bicyclists and transportation planners to improve safety.

## 1. Introduction

Physical activity, such as bicycling, provides health benefits for all ages [1, 2]. Bicycling can reduce the risk of all-cause mortality independent of participation in other types of leisure activity [1, 2]. Despite its potential to improve health, rates of bicycling vary widely by country and between cities [3–6]. Individual-level characteristics (e.g., gender, age, and income), nonmodifiable factors (e.g., climate and geography), and environmental characteristics, including urban design and safety, contribute to these variations and influence whether people choose to bicycle [7, 8]. 

The inherent injury risks associated with bicycling in part explain why more people do not cycle. Only 1.3% of Canadians cycled to work in 2006 [9]. Bicycling injuries are common and result in an important number of emergency department (ED) visits and hospitalizations [10–12]. Bicycling injuries involving motor vehicles (MVs) tend to result in severe injuries and may result in death [10]. Personal characteristics including age (<6 years or >39 years), sex (males), alcohol use, and high speed are known to be associated with severe bicycling injuries [10, 13, 14]. 

To date, the majority of injury risk studies have focused on individual-level risk factors with few taking into consideration upstream determinants of risk such as urban and transportation planning and policy [15]. Creating built environments that are safe and convenient for bicycling as well as other forms of physical activity is an example of an upstream intervention that has the potential to both lower the incidence of injuries and increase levels of physical activity at the population level. While there is a plethora of evidence associating built environmental characteristics with physical activity behaviour [15, 16], limited evidence on the relationship between built environment and injury exists, especially for injuries involving bicyclists. Nevertheless, the available evidence suggests an increased risk of injury associated with bicycling on the sidewalk compared with bicycling off-road paths or trails [17, 18]—a finding which may be due to poor sidewalk maintenance or conflicts with pedestrians. Roundabouts have also been found to be particularly dangerous for bicyclists [19, 20], perhaps due to a high number of potential conflict points or driver distraction and other challenges while navigating the roundabout. Apart from these factors, there is a lack of data on built environmental determinants of bicycling injuries. This area of research has been identified as a priority in order to develop effective place-related interventions for activity promotion and injury prevention [8].

The purpose of this study was to examine the built environmental characteristics (e.g., road/path characteristics, natural features, and obstacles) of locations where bicycling injuries occurred in two urban Alberta cities, by combining data on injury circumstances with street-level environmental audits of crash locations. The objectives were to (1) compare the characteristics of locations where bicyclists were struck by a MV with those of locations where bicyclists were injured in non-MV-related incidents and (2) compare the characteristics of severe injury crash locations with those of nonsevere injury locations.

## 2. Materials and Methods

### 2.1. Study Design and Sample Recruitment

In this case-control study, participants were injured bicyclists of all ages who presented to any one of 7 EDs in Calgary (Alberta Children's Hospital, Foothills Medical Centre, Rockyview Hospital, Peter Lougheed Centre) or Edmonton (Stollery Children's Hospital, University of Alberta Hospital, North East Community Health Centre), Alberta, from May to October 2010. These EDs were chosen as they represent all the hospitals in Calgary and a sufficient number of sites in Edmonton to cover a representative catchment area in that city. In addition, the Foothills Medical Centre, Alberta Children's Hospital, University of Alberta Hospital, and Stollery Children's Hospital are designated adult and paediatric regional trauma centres for their respective areas.

Any injured bicyclist who presented to one of the study EDs was eligible. Eligible patients were identified using the Regional Emergency Department Information System and by reviewing ED records daily. Patients were excluded if they did not speak English, were cycling indoors, using a stationary exercise bicycle, were not riding the bicycle (e.g., cleaning or walking with the bike) at the time of the injury, or if they could not provide sufficient details about the crash location for auditors to visit the site. Bicyclists who were injured outside the established city limits of Calgary or Edmonton were also excluded due to feasibility issues in conducting the audits (i.e., distance). Following informed consent, eligible bicyclists were interviewed in the ED using a questionnaire developed for the study. The questionnaire was based on previous work on bicycle and motorcycle injuries [10, 21], and was pilot tested with a convenience sample of respondents. If bicyclists were missed in the ED, they were mailed a study information package and contacted by telephone. If they consented, a telephone interview was conducted. Participants were asked questions about the circumstances surrounding their crash, including the location, date, and time. Injury information was extracted from the patients' medical chart. 

Two case definitions were used. The first case group included bicyclists injured in a collision with an MV. The second case group included bicyclists whose injuries required admission to a hospital unit after their ED presentation. If a bicyclist had a collision with an MV and was hospitalized, they were included in both case groups. Controls were recruited from the same EDs. A separate control group was selected for each case series. The first control group (for MV cases) was composed of bicyclists injured while riding on the road or the sidewalk but not struck by an MV. Bicyclists riding on the sidewalk were included as potential controls for MV cases because they were exposed to MVs even though they were not riding directly on the road (e.g., when crossing a street). The second control group (for severe cases) included bicyclists with minor injuries, regardless of riding location (e.g., bike paths) or mechanism of injury (MV or other). Cases and controls were individually matched based on time and day of the week, within two weeks prior to or following the case event. Each case was matched to as many as 3 controls; however, MV cases with severe injuries were matched with up to 6 controls (3 MV controls and 3 minor injury controls). We combined data on bicyclists' crash circumstances and injuries with information on the environmental characteristics of the crash locations. 

### 2.2. Environmental Audits of Crash Locations

Street-level data were collected by auditing crash locations using the Systematic Pedestrian and Cyclist Environment Scan (SPACES), which has been demonstrated to have acceptable levels of interrater reliability [22]. Auditors recorded information for both sides of a street segment. Side 1 was defined as the bicyclist's direction of travel. If the direction of travel was unknown, auditors agreed on which side to record as side 1. In general, the area under observation was approximately equal to one street block; however, this varied depending on the location. An additional audit form (see Supplementary Material, available online at doi:10.1155/2012/487681) was created to record features thought to be related to bicycling that were not captured by the SPACES. Trained research assistants (RAs) visited the locations for each matched set at the same time of day as the case injury event, as soon as possible after the event. When resources permitted, two RAs visited the sites and conducted the audits separately in order to assess interrater reliability. One RA was blinded to the case-control status of the location, and their data were used for the analysis. The environmental features examined were divided into six groups including traffic factors (e.g., traffic volume), land use (e.g., types of buildings), path characteristics (e.g., path material), roadway characteristics (e.g., number of lanes), safety features (e.g., surveillance), and aesthetics (e.g., cleanliness).  Table 1 presents a summary of the definitions of environmental characteristics assessed in the audits. 

### 2.3. Statistical Analysis

The characteristics of locations were described by case and control status. Using a complete case analysis, conditional logistic regression was used to examine the effect of each exposure (i.e., 37 environmental characteristics) on the outcomes of interest while controlling for confounders. The number of matched sets in the study limited the number of potential independent variables that could be examined in the conditional logistic regression. It has been suggested that for modeling, the number of independent variables should not exceed 10% of the number of participants in the least frequent outcome category (in this analysis, cases) [23]. Given our matched design, this guideline was applied to the number of discordant sets [24]. It has also been suggested that the “rule” can be relaxed, allowing the researcher to explore more independent variables to assess confounding [25]. We adopted this approach given the importance of considering known individual risk factors for bicycle injuries. Potential confounders were added to the model individually, and if any of the confounders changed the crude estimate by >15% [25], it was retained. If more than one variable changed the estimate by 15%, the one which produced the greatest change was retained. Potential confounders included age, sex, bicycling faster than usual, and self-reported bicyclist speed [10, 26, 27]. Although alcohol use has been shown to be associated with severe bicycling injuries, it was not included as a potential confounder because of its low prevalence in the study sample. “Not applicable” or “unknown” data were treated as missing values and thus were not included in the logistic regression estimates. Adjusted odds ratios (ORs) and corresponding 95% confidence intervals (CIs) were calculated. 

To adjust for multiple confounders simultaneously, we conducted a sensitivity analysis where the matching was unlinked. Unconditional logistic regression was used, and the original matching criteria were added as a variable in the model, in addition to potential confounders. The results of the unmatched analysis were compared with the matched results. Again, we relaxed the 10% “rule” [23], and potential confounders were added to the model following a 5–9 event per variable guideline [24, 25, 28]. 

Ethical approval was granted from the University of Calgary Conjoint Health Research Ethics Board and the University of Alberta Health Research Ethics Board. All patients gave informed consent.

## 3. Results

**Figure 1:**
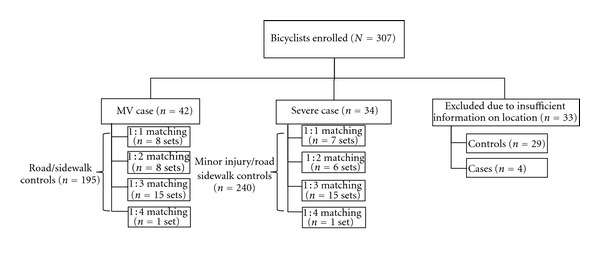
Study sample selection process for matched cases and controls.

In total, 274 injury sites were audited (Figure 1). There were 151 audits conducted in Edmonton, and 123 in Calgary. Six MV cases were also included in the severe case group. Twenty-nine controls and 4 cases were excluded from the study because they did not provide enough details about the crash location for auditors to visit the sites. We chose to report differences of at least 20% in the proportions of either case group and associated controls with a particular feature (Table 2) and estimates that suggested at least a 50% change in the odds of MV collision or severe injury (Tables 3 and 4). None of the estimates suggesting a change less than 50% were statistically significant. 

### 3.1. Characteristics of Case and Control Locations

Table 2 indicates that, compared with controls, a greater proportion of MV case sites had estimated vehicle speeds above 30 km/hr. While the predominant land use was housing, MV case locations occurred more often close to retail establishments. A higher proportion of MV case locations had path obstructions, ≥4 lanes of traffic, or were intersections. Natural features were more prevalent at MV control compared with case sites. A higher proportion of severe injury control sites had sidewalks, roads in good condition, lighting over the path, or high surveillance. A higher proportion of severe case sites had ≥4 lanes of traffic. 

### 3.2. Risk Factors for MV Collisions

From Table 3, in the matched analysis, compared with low traffic volume locations, medium and high volume sites were associated with higher odds of MV collisions. When the volume estimates were adjusted for sex (not shown), the estimated OR for high volume was 2.92 (95% CI [1.07, 7.96]), compared with low volume. The presence of retail or service establishments, path obstructions, parking restrictions, nonmountable curbs, traffic control devices, intersections, or destinations, each significantly increased the odds of being involved in a collision with an MV. The odds of a collision with an MV were lower in locations where natural features were present compared with locations where natural features were absent. 

The results of the unmatched analysis were similar to the conditional logistic regression estimates (Table 3). The estimates for traffic volume, retail, path obstructions, destinations, and natural features were comparable, with some increase in precision demonstrated by narrower confidence intervals. After adjusting for multiple confounders the estimates for services, parking restrictions, curb design, and traffic control devices were no longer statistically significant. 

### 3.3. Risk Factors for Severe Injury (Hospitalization)

The matched analysis results in Table 4 suggest that traffic volume may be related to severe injuries; however, the result was not statistically significant. The presence of a retail establishment increased the odds of severe injury. Locations with a multiuse path had lower odds of severe injury compared with locations with a sidewalk. Good road conditions, compared with poor road condition, lowered the odds of severe injury. Nonmountable curbs increased the odds of severe injury. 

The unmatched analysis results were similar to the matched results. The presence of retail land use remained an important predictor of severe injuries, while good road condition still reduced the odds of severe injury. The presence of street lighting and high surveillance from surrounding buildings both had a point estimate that indicated a reduction in the odds of a severe injury. 

### 3.4. Audit Data Interrater Reliability

Ninety-seven locations were audited by two observers. Interrater agreement was generally high (≥95%); most items had a 1%-2% difference in responses. Items with ≥5% differences between raters included path condition, slope, and obstructions. For land use, path, and roadway characteristics, Kappa (*κ*) ranged from 0.3 for presence of offices and cleanliness to 0.9 for schools and number of lanes; overall, 78% of items had at least substantial agreement (*κ* ≥ 0.61). For MV cases the proportion of items with substantial agreement was 60%, compared with 73% for controls. For severe cases and minor injury controls, 76% of items had substantial agreement.

## 4. Discussion

This study provides a comprehensive description of the locations where bicycling injuries occurred, bringing attention to built environmental features that increase the likelihood of a bicyclist-MV collision or severe injury. Our results show that traffic volume is a significant risk factor for bicycle-MV collisions. Medium and high volume presented at least a twofold increase in the odds of collision compared with low volume roads. At intersections the odds of collision were much higher than at nonintersection locations. Good road conditions were associated with a reduction in the odds of severe injury. When we examined the odds ratio estimates associated with various types of land use, bicyclists were more at risk near retail establishments. Some aesthetic and safety items, such as streetlights and high surveillance showed a 30%–40% reduction in the odds of severe injury. We examined the effect of several potential confounders previously shown to be related to bicycling injuries: age, sex, self-reported bicyclist speed, bicycling faster than usual, and time of day [10, 12, 29]. In both the matched and unmatched analyses, adjustment for age had the greatest impact on the magnitude of the odds ratio estimates between the environmental characteristics and injury. This is consistent with other research where age has been identified as one of the major risk factors for injury in bicyclists [10, 11]. 

The estimates for high traffic volume were lower than those for medium volume, which may be related to road configuration. High volume roads might be thoroughfares where traffic flow is uninterrupted (e.g., highways), creating fewer opportunities for bicyclists and vehicles to cross paths. Alternatively, medium volume locations might be areas with many intersections, presenting more opportunities for encounters. While few studies have directly examined the link between traffic volume and bicyclist injury, it has been shown that roads designed to accommodate more traffic, such as arterial and divided roads, increase the overall risk of bicycle-vehicle collisions [30, 31]. 

Significantly lower odds of severe injury were observed for locations with multiuse paths compared with sidewalks in the matched analysis. Bicycle paths are reported to be favoured by many and have been linked to increased bicycling [32]. Other studies have found an increased risk of injury on sidewalks compared with off-road paths [17, 18, 33], a reduced risk of injury on paths compared with the road [34] and reduced odds of fatality or hospitalization when bicycling on a facility other than the road such as sidewalks, driveways, or multiuse paths [11, 29]. This growing evidence suggests that separating bicyclists from pedestrians and MVs with facilities like dedicated bicycling lanes can reduce the risk of injury. 

For land use, the presence of retail establishments increased the odds of MV collisions and severe injuries. It is likely that these commercial sites have more traffic volume, population density, intersections, and distractions, creating opportunities for bicyclist-MV encounters. The relationship between commercial establishments and bicyclist-MV crashes has been found elsewhere with the presence of strip malls and big box stores (e.g., Wal-Mart), which were associated with an increased number of bicyclist-MV encounters compared with noncommercial locations [31]. This suggests that safety gains could be made by diverting bicyclists away from heavy traffic in commercial areas (e.g., parking lots), perhaps by locating parking lots at the rear of shops or by providing designated bicycle path access to shops. 

Streetlights and surveillance were associated with significant reductions in the odds of severe injury. It may be that driving behaviour is influenced by having “eyes on the street” (e.g., people drive slower when they are aware of people watching them), resulting in fewer or less severe bicycling injuries. Alternatively, traffic volume may be higher in areas with less surveillance (e.g., arterial roads). While few studies have examined safety or aesthetic features and injuries together, some studies have examined the effect of street lighting. These studies have shown that severe injuries are more likely in unlit areas, and that lighting reduces injuries in rural areas [26, 35, 36]. It is important to keep this evidence in mind, especially in locations like Alberta where it is dark early in the morning and late in the afternoon for much of the year, which correspond to commuting times. This is an important finding from a planning perspective and suggests that more street lighting or better light coverage for roadways and pathways could help prevent injuries. 

When comparing the factors related to MV and severe injury outcomes we found that factors related to one outcome were not necessarily related to the other. Recognizing that features of the environment associated with MV collisions and severe injuries may differ from one another is important for planners, as it signals that multiple design elements need to be considered in order to reduce the occurrence of each type of event. Focusing on the factors that are identified as predictors of both outcomes is crucial. 

### 4.1. Limitations

This study has some limitations that require discussion. We collected information on potential confounders; however, it is possible that factors we did not anticipate or collect information on could be independently related to the outcomes and the characteristics we examined. One such possible confounder is bicyclist volume. While we did count bicyclists, the majority of the sites we visited had low/no bicyclist volume. Given the lack of information on this factor, it was not included in the adjusted analysis. It has been suggested that greater bicyclist volume is associated with a reduction in the number of injuries (i.e., the “safety in numbers” effect); however, a causal relationship has not been established [4, 37, 38]. We did examine bicyclist volume as an independent predictor of MV events and severe injuries and did not find evidence of an association.

To determine the audit sites, we relied on the participant's description of the crash location. Some patients had trouble remembering the exact crash site or their direction of travel at the time. The information they provided is subject to limitation of recall, especially if the patient was interviewed sometime after the event. However, the average time between the injury and the audit date was 48 days, and nearly 80% of audits were conducted within 2 months of the injury date. Many interviews were conducted by telephone, and resources did not permit us to provide maps for patients to pinpoint their crash site.

Bicyclists who could not provide sufficient details about the crash location for it to be accurately identified were excluded from the analysis. If these bicyclists differed systematically from those included in the sample, it could introduce selection bias. To examine this potential bias, the excluded bicyclists were compared with the overall study sample. Few differences were detected between the two groups; compared with the study sample, a slightly higher proportion of excluded bicyclists were 13–17 years old, and they had a lower proportion of helmet use. Because of these minor differences, it may be that there were fewer adolescents in our sample than would have been included otherwise. Adjustment for age in the analysis would have eliminated this concern. In addition, because excluded bicyclists made up a very small proportion of the overall sample, their exclusion is unlikely to have had any major effect on the findings.

While there is potential for misclassification of the built environment characteristics of audited locations, when we examined the reliability of the audit data from locations where two observers completed observations, there was no indication that items were inconsistently recorded. Kappa values indicated “substantial” to “almost perfect” agreement for the majority of items, and differences in agreement between case and control locations were minor. In addition, we used the data from the blinded auditor for all analyses, reducing the likelihood of observer bias. 

## 5. Conclusion

The built environment cannot be overlooked in injury prevention strategies. Creating safe, activity friendly environments is vital to encouraging higher levels of physical activity participation [8], especially considering the obesity epidemic and the shift towards encouraging eco-friendly transportation. Our findings point to specific built environmental characteristics including traffic volume, land use, path designs, and roadway features as risk factors for MV collisions or severe injuries. This information should provide urban and transportation planners with robust evidence upon which to base decisions regarding environments that are safe and conducive to bicycling. Furthermore, by disseminating this information to end users, bicyclists will be aware of the dangers posed by certain features, enabling them to make safe route choices. 

## Supplementary Material

The additional environmental audit form was developed by the researchers to capture additional potential risk factors for bicycle-related injuries that were part of the primary audit instrument. This form also provided an area for auditors to record any comments they felt needed to be noted about the location.

## Figures and Tables

**Table 1:** Definitions of environmental characteristics assessed in audits of bicycling injury locations.

Variable	Definition
Traffic speed	≤30 km/hr versus >30 km/hr estimated average vehicle speed.
Traffic volume	Number of vehicles per hour; 3 categories: (1) low (≤250 vehicles/hr); (2) medium (250–749 vehicles/hr); (3) high (≥750 vehicles/hr).
Bicyclist volume	No bicyclists observed at location versus at least one bicyclist.
Path type	Sidewalk, multiuse path (shared by bicyclists and pedestrians) with markings (e.g., center line, stencils), or without markings.
Path location	For each side 3 categories: (1) within 1 metre of roadway; (2) between 1 and 3 metres; (3) >3 metres.
Path material	For each side, continuous or slab concrete and bitumen (asphalt) versus gravel and grass.
Path/road slope	Flat versus moderate or steep slope (for each side of path).
Path/road condition	Good versus moderate, poor, or under repair (for each side of path).
Path obstructions	For each side, presence of permanent obstructions (poles, signs, trees, benches, tables, fences) versus none.
Bike lane	Marked designated bicycle lane on roadway versus no marked lane.
Roadway lanes	1–3 lanes versus 4 or more lanes.
Curb	For each side 3 categories: (1) mountable curb; (2) nonmountable; (3) no curb. Reference category was mountable curb.
Traffic control devices	Presence of traffic control devices (roundabout, speed bump, chicanes/chokers, lane narrowing, and signals) versus no traffic controls.
Crossing	Presence of crossings (zebra, signals, bridge) versus no crossings.
Crossing aids	Presence of crossing aids (median, kerb extension) versus no crossing aids.
Other routes	Presence of alternate routes (lanes, path through park, no through road) versus no alternate route.
Intersections	Path-path and path-road intersection versus no intersection.
Streetlights	For each side, presence of street lighting versus no street lights.
Lighting on path	For each side, whether lighting covered the path or not.
Destinations	The location provided access to services or other destinations (e.g., park, convenience store, businesses) versus no destinations.
Surveillance	Location could be observed from ≥75% versus less than 75%.
Maintenance	Location gardens and verges were >75% well maintained versus less than 75%.
Verge trees	Presence of tress along the verge versus no trees.
Tree height	Tall or medium sized trees versus small trees.
Cleanliness	Location is clean (free of debris, garbage, graffiti, etc.) versus some uncleanliness.
Natural features	Presence of parks, green space, river, lakes, and so forth.
Path width	Path is 150 centimetres or less versus wider than 150 centimetres.
Age	≤14 years old versus ≥15 years old.
Bicyclist speed (“speed”)	<15 km/hr versus ≥15 km/hr.
Bicycling faster than usual (“riding fast”)	Bicyclist reported “cycling faster than usual” at the time of the incident.
Peak time	Peak time (Monday–Friday 06:31–08:30 and 16:01–18:00) versus off-peak time and weekends (Saturday and Sunday)

**Table 2:** Bicycle crash location characteristics by case and control groups.

	MV controls *n* = 195	(%)	MV cases *n* = 42	(%)	Severe controls *n* = 240	(%)	Severe cases *n* = 34	(%)
Traffic and land use characteristics								
Estimated avg. speed > 30 km/hr	121	(62.1)	35	(83.3)	134	(55.8)	22	(64.7)
n/a	8	(4.1)	0	(0.0)	38	(15.8)	7	(20.6)
Unknown	25	(12.8)	1	(2.4)	25	(10.4)	1	(2.9)
Predominant feature								
Housing	114	(58.5)	17	(40.5)	122	(50.8)	15	(44.1)
Retail	7	(3.6)	10	(23.8)	12	(5.0)	5	(14.7)
Nature	34	(17.4)	4	(9.5)	58	(24.2)	6	(17.6)

Path characteristics (side 1)								
Path type								
No path	19	(9.7)	5	(11.9)	21	(8.8)	3	(8.8)
Sidewalk	137	(70.3)	30	(71.4)	154	(64.2)	20	(58.8)
Shared with markings	11	(5.6)	1	(2.4)	26	(10.8)	6	(17.6)
Shared no markings	28	(14.4)	6	(14.3)	39	(16.3)	5	(14.7)
Path location								
Within 1 m of road	114	(58.5)	30	(71.4)	128	(53.3)	16	(47.1)
Btw 1 and 3 m of road	33	(16.9)	6	(14.3)	33	(13.8)	7	(20.6)
>3 m from road	28	(14.4)	1	(2.4)	54	(22.5)	8	(23.5)
n/a	19	(9.7)	5	(11.9)	21	(8.8)	3	(8.8)
Unknown	1	(0.5)	0	(0.0)	4	(1.7)	0	(0.0)
Good condition	111	(56.9)	20	(47.6)	135	(56.3)	15	(44.1)
n/a	19	(9.7)	5	(11.9)	21	(8.8)	3	(8.8)
Any obstructions	79	(40.5)	22	(52.4)	103	(42.9)	13	(38.2)
n/a	19	(9.7)	5	(11.9)	21	(8.8)	3	(8.8)

Path characteristics (side 2)								
Path type								
No path	62	(31.8)	8	(19.0)	68	(28.3)	7	(20.6)
Sidewalk	110	(56.4)	26	(61.9)	128	(53.3)	14	(41.2)
Shared with markings	4	(2.1)	1	(2.4)	13	(5.4)	4	(11.8)
Shared no markings	16	(8.2)	6	(14.3)	24	(10.0)	4	(11.8)
n/a	3	(1.5)	0	(0.0)	6	(2.5)	5	(14.7)
Unknown	0	(0.0)	1	(2.4)	1	(0.4)	0	(0.0)
Path location								
Within 1 m of road	80	(41.0)	25	(59.5)	93	(38.8)	12	(35.3)
Btw 1 and 3 m of road	32	(16.4)	8	(19.0)	36	(15.0)	4	(11.8)
>3 m from road	17	(8.7)	0	(0.0)	32	(13.3)	6	(17.6)
n/a	65	(33.3)	8	(19.0)	74	(30.8)	12	(35.3)
Unknown	1	(0.5)	1	(2.4)	5	(2.1)	0	(0.0)
Good condition	85	(43.6)	21	(50.0)	103	(42.9)	13	(38.2)
n/a	65	(33.3)	8	(19.0)	74	(30.8)	12	(35.3)
Any obstructions	48	(24.6)	19	(45.2)	64	(26.7)	9	(26.5)
n/a	65	(33.3)	8	(19.0)	74	(30.8)	12	(35.3)

Roadway characteristics								
Marked bike lane	8	(4.1)	4	(9.5)	11	(4.6)	1	(2.9)
n/a	0	(0.0)	0	(0.0)	30	(12.5)	7	(20.6)
Unknown	3	(1.5)	1	(2.4)	3	(1.3)	1	(2.9)
Good road condition	132	(67.7)	25	(59.5)	144	(60.0)	13	(38.2)
n/a	0	(0.0)	0	(0.0)	30	(12.5)	7	(20.6)
>4 lanes of traffic	62	(31.8)	23	(54.8)	70	(29.2)	15	(44.1)
n/a	0	(0.0)	0	(0.0)	30	(12.5)	7	(20.6)
Unknown	4	(2.1)	0	(0.0)	4	(1.7)	0	(0.0)
Mountable curb (side 1)	115	(59.0)	22	(52.4)	123	(51.3)	14	(41.2)
n/a	19	(9.7)	2	(4.8)	50	(20.8)	8	(23.5)
Unknown	2	(1.0)	0	(0.0)	2	(0.8)	0	(0.0)
Traffic control devices	130	(66.7)	18	(42.9)	133	(55.4)	15	(44.1)
n/a	0	(0.0)	0	(0.0)	30	(12.5)	7	(20.6)
Missing	4	(2.1)	0	(0.0)	2	(0.8)	2	(5.9)
Intersections	118	(60.5)	36	(85.7)	152	(63.3)	22	(64.7)
Unknown	6	(3.1)	0	(0.0)	88	(36.7)	12	(35.3)

Safety characteristics								
Lights over path (side 1)	114	(58.5)	27	(64.3)	129	(53.8)	15	(44.1)
n/a	47	(24.1)	10	(23.8)	75	(31.3)	12	(35.3)
Unknown	1	(0.5)	0	(0.0)	3	(1.3)	0	(0.0)
Lights over path (side 2)	106	(54.4)	30	(71.4)	126	(52.5)	13	(38.2)
n/a	46	(23.6)	5	(11.9)	68	(28.3)	12	(35.3)
Unknown	1	(0.5)	0	(0.0)	4	(1.7)	0	(0.0)
Destinations	92	(47.2)	30	(71.4)	116	(48.3)	17	(50.0)
Unknown	0	(0.0)	0	(0.0)	1	(0.4)	0	(0.0)
High surveillance	101	(51.8)	19	(45.2)	116	(48.3)	10	(29.4)
n/a	5	(2.6)	0	(0.0)	19	(7.9)	5	(14.7)

Aesthetic characteristics								
Natural features	105	(53.8)	14	(33.3)	133	(55.4)	22	(64.7)
Attractive for cycling	169	(86.7)	32	(76.2)	208	(86.7)	27	(79.4)
Difficult for cycling	48	(24.6)	14	(33.3)	66	(27.5)	12	(35.3)
Continuity	172	(88.2)	39	(92.9)	210	(87.5)	33	(97.1)
n/a	1	(0.5)	0	(0.0)	1	(0.4)	0	(0.0)
Unknown	7	(3.6)	0	(0.0)	7	(2.9)	0	(0.0)

**Table 3:** Matched and un-matched logistic regression estimates of the association between environment risk factors and MV/bicyclist collisions.

	Matched OR	95% CI	Adjustment factors	Un-matched OR	95% CI	Adjustment factors
Traffic volume						
Low^a^	1	Reference	^ b^	1	Reference	^ b^
Med^a^	5.13*	1.44, 18.27	Riding fast	3.49*	1.37, 8.88	Age
High^a^	2.34	0.75, 7.24	Riding fast	2.83*	1.24, 6.42	Age
High speed limit (>30 km/hr)	3.18	0.62, 16.41	^ b^	2.59	0.87, 7.71	^ b^

Land use						
Offices	8.8	0.99, 78.16	^ b^	3.01	0.73, 12.36	Age, day/time and riding fast
Retail	5.56*	1.72, 17.98	Age	7.54*	3.15, 18.03	Age and speed
Industry	2.07	0.28, 15.43	^ b^	3.70	0.48, 28.82	Age and speed
Services	3.80*	1.29, 11.69	^ b^	2.02	0.96, 4.23	Age
Nature	0.38	0.14, 1.00	Speed	0.44*	0.21, 0.91	^ b^

Path characteristics (side 1)						
Type of path						
Sidewalk	1	Reference		1	Reference	
No path	1.66	0.14, 19.75	Age	1.30	0.43, 3.89	Speed
Multi-use path^#^	0.63	0.16, 2.51	Age	1.05	0.41, 2.68	Speed
Path location (distance from road)						
Within 1 m	1	Reference		1	Reference	
Btw 1 and 3 m	1.02	0.26, 3.95	Sex	0.40	0.14, 1.12	Age and day/time
>3 m	0.17	0.02, 1.53	Age	0.16	0.02, 1.29	Age and day/time
Sloped path	1.60	0.35, 7.36	^ b^	1.14	0.35, 3.71	Riding fast
Path obstructions	1.05	0.38, 2.93	Age	1.80	0.88, 3.70	^ b^

Path characteristics (side 2)						
Type of path						
Sidewalk	1	Reference		1	Reference	
No path	0.38	0.12, 1.26	Riding fast	0.68	0.28, 1.68	Age and speed
Multi-use path^#^	0.61	0.10, 3.78	Riding fast	1.69	0.59, 4.85	Age and speed
Sloped path	0.15	0, 1.99	^ b^	0.72	0.18, 2.98	Day/time, riding fast and speed
Path obstructions	3.83*	1.03, 14.25	^ b^	2.59*	1.13, 5.90	Speed

Roadway characteristics						
Designated bike lane	0.64	0.10, 4.19	^ b^	1.83	0.42, 8.02	Day/time, riding fast and age
Parking restrictions	3.88*	1.31, 11.55	^ b^	1.74	0.85, 3.55	Age
Curb						
Mountable	1	Reference		1	Reference	
Not mountable	3.03*	1.04, 8.82	Sex	1.35	0.65, 2.82	Riding fast
No curb	0.84	0.13, 5.59	Sex	0.55	0.12, 2.56	Riding fast
Curb cuts	2.71	0.84, 8.72	^ b^	0.82	0.38, 1.78	Riding fast and age
Traffic control devices	2.74*	1.02, 7.35	Riding fast	1.59	0.74, 3.45	Age and day/time
Intersection	6.89*	1.48, 32.14	^ b^	2.83*	1.11, 7.20	Age
Crossings	1.68	0.62, 4.54	^ b^	1.54	0.73, 3.26	Age and day/time
Crossing aids	0.4	0.10, 1.52	^ b^	0.43	0.16, 1.18	Age and speed

Safety characteristics						
Street lights (side 2)	2.38	0.51, 10.99	^ b^	1.54	0.43, 5.58	Age
Lighting over path (side 2)	1.03	0.30, 3.53	^ b^	1.70	0.69, 4.16	^ b^
Destinations	2.4	1.01, 6.00*	Sex	2.35*	1.11, 4.97	Riding fast
Aesthetic characteristics						
>1 tree/block (side 1)	3.25	0.37, 28.46	^ b^	1.71	0.63, 4.63	Riding fast
Tall trees (side 1)	2.27	0.26, 20.06	^ b^	1.08	0.21, 5.45	Riding fast
Natural views	0.2*	0.07, 0.69	Age	0.43*	0.21, 0.86	^ b^
Difficult for bicycling	2.00	0.68, 5.92	^ b^	1.92	0.55, 6.66	Speed and day/time

MV: motor vehicle; OR: odds ratio; CI: confidence interval.

*Represents significance based on CI not including the null value.

^a^Low volume: ≤250 vehicles/hr, medium volume: 250–749 vehicles/hr, high volume: ≥750 vehicles/hr.

^b^If model could not accommodate the addition of one or more covariates or there was no evidence of confounding, the crude estimate was retained.

**Table 4:** Matched and un-matched logistic regression estimates of the association between environment risk factors and severe bicyclist injury.

	Matched OR	95% CI	Adjustment factors	Un-matched OR	95% CI	Adjustment factors
Traffic volume						
Low^a^	1	Reference		1	Reference	
Med^a^	3.20	0.63, 16.25	Riding fast	1.22	0.37, 4.01	Age
High^a^	2.01	0.51, 7.94	Riding fast	1.53	0.61, 3.85	Age
Avg. traffic speed (>30 km/hr)	1.7	0, 3.91	^ b^	4.13	0.89, 19.11	Speed and riding fast
High speed limit (>30 km/hr)	1.85	0.46, 7.47	^ b^	1.14	0.41, 3.21	^ b^

Land use						
Retail	8.12*	1.66, 39.7	^ b^	2.53*	1.10, 5.83	Age
School	0.24	0.03, 1.05	^ b^	0.35	0.04, 2.84	Age and speed
Nature	0.41	0.14, 1.24	Age	1.25	0.60, 2.57	^ b^

Path characteristics (side 1)						
Type of path						
Sidewalk	1	Reference		1	Reference	
No path	1.29	0.24, 6.81	Riding fast	1.10	0.30, 4.02	^ b^
Multi-use path^#^	0.24*	0.08, 0.77	Riding fast	1.30	0.59, 2.87	^ b^
Path location (distance from road)						
Within 1 m of road	1	Reference		1	Reference	
Btw 1 and 3 m of road	0.54	0.14, 2.09	Age	1.68	0.59, 4.81	Speed
>3 m from road	0.48	0.16, 1.45	Age	1.42	0.52, 3.85	Speed
Sloped path	0.42	0, 1.63	^ b^	1.83	0.71, 4.76	Speed
Path obstructions	0.36	0.13, 1.01	Age	0.81	0.38, 1.74	^ b^

Path characteristics (side 2)						
Type of path						
Sidewalk	1	Reference		1	Reference	
No path	1.14	0.36, 3.62	Age	0.86	0.28, 2.65	Age and speed
Multi-use path^#^	0.40	0.10, 1.68	Age	2.12	0.73, 6.21	Age and speed
Path location (distance from road)						
Within 1 m	1	Reference		1	Reference	
Btw 1 and 3 m	0.11	0.01, 1.64	Age	0.96	0.28, 3.31	Speed
>3 m	0.36	0.06, 2.06	Age	1.95	0.59, 6.49	Speed
Sloped path	1.11	0.23, 5.32	^ b^	1.93	0.65, 5.75	^ b^
Path obstructions	3.32	0.65, 16.9	^ b^	1.10	0.45, 2.73	^ b^

Roadway characteristics						
Good road condition	0.25*	0.07, 0.96	^ b^	0.43*	0.19, 0.96	^ b^
>4 lanes of traffic	2.59	0.80, 8.4	^ b^	2.31	0.87, 6.16	Age, speed, and riding fast
Curb						
Mountable	1	Reference		1	Reference	
Non-mountable	4.51*	1.08, 18.8	Sex	1.62	0.71, 3.71	^ b^
No curb	0.34	0.04, 3.17	Sex	0.44	0.05, 3.53	^ b^
Crossings	1.1	0.39, 3.15	^ b^	1.85	0.75, 4.58	Speed
Other routes	0.44	0.16, 1.16	Riding fast	0.75	0.34, 1.68	Speed

Safety characteristics						
Street lights (side 1)	1.90	0.70, 5.12	Age	0.78	0.34, 1.79	Speed
Street lights (side 2)	0.89	0.29, 2.77	^ b^	0.38*	0.15, 0.97	Age and speed
Lighting over path (side 1)	0.36	0.07, 1.86	^ b^	0.43	0.15, 1.22	Sex
Lighting over path (side 2)	0.58	0.16, 2.15	^ b^	0.39	0.15, 1.04	Sex
High surveillance	0.53	0.17, 1.71	^ b^	0.32*	0.13, 0.82	Speed
Aesthetic characteristics						
>1 tree/block (side 1)	2.19	0.41, 11.78	^ b^	2.07	0.57, 7.53	Speed
>1 tree/block (side 2)	1.98	0.22, 17.88	^ b^	1.75	0.48, 6.42	Speed
Clean	1.79	0.59, 5.43	Age	1.52	0.60, 3.85	^ b^
Attractive for bicycling	0.4	0.13, 1.23	^ b^	0.49	0.18, 1.32	Age and speed
Continuity of path	3.0	0.35, 25.96	^ b^	2.34	0.29, 19.04	Age, speed, and riding fast

MV: motor vehicle; OR: odds ratio; CI: confidence interval.

*Represents significance based on CI not including the null value.

^a^Low volume: ≤250 vehicles/hr, medium volume: 250–749 vehicles/hr, high volume: ≥750 vehicles/hr.

^b^If model could not accommodate the addition of one or more covariates, the crude estimate was retained.

## Abbreviations

ED: Emergency department
MV: Motor vehicle
OR: Odds ratio
CI: Confidence interval.
