# Supplementary material for: Environmental Determinants of Bicycling Injuries in Alberta, Canada
Source: J Environ Public Health. 2012 Nov 28;2012:487681. doi: 10.1155/2012/487681 (PMC3515916; doi:10.1155/2012/487681)
Supplement: Supplementary file 1 — The additional environmental audit form was developed by the researchers to capture additional potential risk factors for bicycle-related injuries that were part of the primary audit instrument. This form also provided an area for auditors to record any comments they felt needed to be noted about the location. [file 487681.f1.pdf]

Appendix A

Environmental Determinants of Cycling Injuries: Additional environmental audit

Observer:__________________ Control site ☐ Calgary ☐
Case site☐ Edmonton ☐

Date: _______ / _______/ ______
Day Month Year

Location description: __________________________ _

A. Path for walking &/or cycling
36. Path width (cm):_______________
5a. Intersection:
N/A ☐
Path/path ☐
Path/road ☐
Path/other:__________________

5b. Kerb cuts:
N/A ☐
Kerb lip height (cm):____________

21a. Parking:
N/A ☐
On street parallel parking ☐
On street angle parking ☐
Parking lot (fill out 21) ☐
Other: _______________________

Comments:
Please write the question number, if the comment related to a specific item on the SPACES tool.

____
____
____
____
____

To be filled out from the cyclist interview if location is a case site only:

Type of error (check one only):
Mid-block ride out ☐
Cyclist inattention/error ☐
Driver inattention/error ☐
Other:____
